# Supplementary material for: Rediversification Following Ecotype Isolation Reveals Hidden Adaptive Potential
Source: bioRxiv. 2023 May 4:2023.05.03.539206. Preprint. [Version 1] doi: 10.1101/2023.05.03.539206 (PMC10187175; doi:10.1101/2023.05.03.539206)
Supplement: Supplement 1 [file media-1.pdf]

Rediversification Following Ecotype Isolation Reveals Hidden Adaptive Potential

Supplementary Information

Joao A Ascensao, Jonas Denk, Kristen Lok,
QinQin Yu, Kelly M Wetmore, Oskar Hallatschek

2023

Contents

1 Table of strains **2**

2 Table of plasmids **3**

3 Table of oligonucleotides **4**

4 Mutations in S_B clones relative to their ancestor **4**

4.1 6.5k S_B 1 5
4.2 6.5k S_B 2 5
4.3 6.5k S_B 4 5
4.4 6.5k S_B 5 5
4.5 6.5k S_B 6 6
4.6 17k S_B 1 6

List of Figures

S1 Flow cytometry accuracy 8
S2 Flow cytometry controls and bias of S/L CFUs 9
S3 Example of raw flow cytometry data and gates 10
S4 Varying dilution rate and flow rate in the flow cytometer 11

S5 Rediversification of S into S_B 12
S6 Relative fitness of S_B in LB 13
S7 Growth dynamics over 24-hour cycle 14
S8 Heatmap and clustering of gene expression patterns 15
S9 Volcano plot of differential expression 16
S10 Kegg gene set enrichment analysis for S_B clones 16
S11 Comparison of differentially expressed KEGG pathways between S_B and L 17
S12 Gene set enrichment analysis plots, comparing L and S 18
S13 Gene set enrichment analysis plots, comparing S_B and S 19

1 Table of strains

Strain Name	Internal Name	Ancestor	Notes
REL606	REL606		ara- LTEE ancestor
6.5k S 1	eJA046	REL11555	subclone
6.5k S 2	eJA047	REL11555	subclone
6.5k S 3	eJA048	REL11555	subclone
6.5k L 1	eJA027	REL11556	subclone
6.5k L 2	eJA028	REL11556	subclone
6.5k S_B 1	eJA049	6.5k S 1	Rediversified in DM25
6.5k S_B 2	eJA036	6.5k S 1	Sister to 6.5k S_B 4*
6.5k S_B 3	eJA395	6.5k S 1	
6.5k S_B 4	eJA037	6.5k S 1	Sister to 6.5k S_B 2*
6.5k S_B 5	eJA034	6.5k S 2	
6.5k S_B 6	eJA035	6.5k S 3	Rediversified in DM25
17k S 1	eJA052	REL11557	
17k L 1	eJA031	REL11578	
17k S_B 1	eJA055	17k S 1	
17k S_B 2	eJA397	17k S 1	
17k S_B 3	eJA398	17k S 1	
40k S 1	eJA172	REL10927	Isolated from mixed population
40k L 1	eJA174	REL10927	Isolated from mixed population
40k S_B 1	eJA177	40k S 1	
40k S_B 2	eJA399	40k S 1	
40k S_B 3	eJA400	40k S 1	

*6.5k S_B 2 and 4 were isolated from the same rediversification experiment culture–they come from two different colonies from the same timepoint from the same plate.

2 Table of plasmids

Plasmid Name	Description	Source of parts	Source
pBad-EBFP2	Plasmid with eBFP2 gene		[1]; Addgene 14891
pMRE-Tn7-133	miniTn7 plasmid to insert sYFP2 into genome at attTn7		[2]; Addgene 118551
pJA17	miniTn7 plasmid to insert eBFP2 (with strong promoter) into genome at attTn7	Backbone from pMRE-Tn7-133; eBFP2 from pBad-EBFP2; BBa_J23119 promoter [3]; RBS designed in silico [4]	This study
pJA18	miniTn7 plasmid to insert sYFP2 (with strong promoter) into genome at attTn7	Backbone from pMRE-Tn7-133; eBFP2 from pMRE-Tn7-133; BBa_J23119 promoter [3]; RBS designed in silico [4]	This study

3 Table of oligonucleotides

Oligo Name	Sequence	Purpose
ja35	GAAGAGGAT AAAACCGTGGA	Amplify arcA for genotyping
ja36	AGGTCAGGG ACTTTTGTAC	Amplify arcA for genotyping
ja40	TTCGAAGCG ACAGATGGC	Sanger sequence arcA for genotyping
ja37	CAGTTGTGACATA CAGCTAACGCT	Amplify aspS for genotyping
ja38	GCTCATGGGAGTT CACTCAGTTG	Amplify aspS for genotyping
ja42	ACTCTAACC ACGTCAACACC	Sanger sequence aspS for genotyping
ja200	ATTCGCTAA ACTGTgctagcatta tacctaggactgagct agctgtcaagctgtc cataaaaccgccc	Gibson assembly primer to make pJA17/18
ja201	atgctagcACAGTTT AGCGAATACGT CATAGAGCATTA AGGAGGTCATAG atggtgagcaagggcgag	Gibson assembly primer to make pJA17/18
ja183	tccaagctcagctaattacttg tacagctcgtccatgc	Gibson assembly primer to make pJA17
ja184	cgagctgtacaagtaattagct gagcttggactcc	Gibson assembly primer to make pJA17
ja190	cttcgccgatcaggatgcg	Downstream of glmS - amplify attTn7 junction with FP
ja191	tcgccctcgaacttcacctc	In pJA17/18 - amplify attTn7 junction with FP
ja170	caaaatcggttacggttgag	Downstream of glmS - sequence attTn7 junction with FP

4 Mutations in S_B clones relative to their ancestor

All comparison tables generated with (1) `minimap2` [5] and `sniffles` [6] for structural variants, and (2) `breseq` [7] for all other variants.

4.1 6.5k S_B 1

position	mutation	annotation	gene
242,204	(G)9→10	pseudogene (194/373 nt)	ECB_00212 →
942,720	(C)7→8	intergenic (+464/+235)	clpA → / ← serW
1,292,775	A→G	intergenic (-48/-556)	hns ← / → tdk
1,368,422	(T)8→9	intergenic (+40/-172)	pspE → / → ycjM
1,444,298	C→T	R136R (CGC→CGT)	ydbC →
1,829,779	G→A	intergenic (+11/+82)	pncA → / ← ydjE
2,034,188	A→G	Y26Y (TAT→TAC)	manC ←
2,165,552	(C)5→6	pseudogene (306/1685 nt)	yehU ←
2,704,655	T→C	F41F (TTT→TTC)	ygaX →
2,771,495	T→C	T51A (ACC→GCC)	cysN ←
2,998,766	G→A	intergenic (+40/-66)	yqgA → / → pheV
3,937,360	A→G	D43G (GAC→GGC)	rffT →

4.2 6.5k S_B 2

position	mutation	annotation	gene
752,223	C→T	intergenic (+723/-124)	ybgG → / → cydA
942,720	(C)7→8	intergenic (+464/+235)	clpA → / ← serW
1,186,311	G→A	L754F (CTT→TTT)	mfd ←
2,195,273	(C)9→8	coding (110/837 nt)	yeiG →
2,762,201	+T	coding (875/1365 nt)	ygbN →

4.3 6.5k S_B 4

position	mutation	annotation	gene
752,223	C→T	intergenic (+723/-124)	ybgG → / → cydA
942,720	(C)7→8	intergenic (+464/+235)	clpA → / ← serW
1,186,311	G→A	L754F (CTT→TTT)	mfd ←
1,995,685	(C)8→9	intergenic (+103/+351)	yeeN → / ← asnW
2,004,021	-1258 bp	1258 bp deletion	
3,213,279	A→G	A20A (GCA→GCG)	yhaV →
4,598,479	T→C	intergenic (+28/+279)	nadR → / ← yjjK

4.4 6.5k S_B 5

position	mutation	annotation	gene
242,204	(G)9→10	pseudogene (194/373 nt)	ECB_00212 →
1,422,705	INV	179,806 bp inversion	
2,567,537	T→C	L658L (CTA→CTG)	pbpC ←
4,606,996	C→T	A151V (GCC→GTC)	creC →

4.5 6.5k S_B 6

position	mutation	annotation	gene
508,855	A→G	G32G (GGA→GGG)	glxR →
751,910	IS1 (–) +8 bp	intergenic (+410/-430)	ybgG → / → cydA
1,038,663	A→G	V141V (GTA→GTG)	yccR →
1,140,567	+771 bp	771 bp insertion	
1,422,704	INV	179,807 bp inversion	
1,184,045	A→G	R312R (CGT→CGC)	ycfS ←
1,480,910	(A)8→7	coding (786/1407 nt)	ydcR →
2,178,109	G→A	S140L (TCG→TTG)	yohF ←
3,726,210	T→C	intergenic (-70/+10)	cysE ← / ← gpsA
4,288,802	A→G	intergenic (-24/+35)	alsB ← / ← rpiR

4.6 17k S_B 1

position	mutation	annotation	gene
794,722	(G)10→9	pseudogene (282/462 nt)	ECB_00735 →
1,292,373	IS1 (–) +9 bp	coding (346-354/414 nt)	hns ←
1,603,015	T→C	I90V (ATC→GTC)	ECB_01505 ←
1,680,075	+770 bp	770 bp insertion	
1,796,607	(C)10→9	intergenic (-53/+55)	celF ← / ← celD
2,032,376	G→A	T405T (ACC→ACT)	manB ←
2,104,764	(CCAG)21→20	pseudogene (238-241/272 nt)	ECB_01992 →
4,447,544	+34 bp	pseudogene (636/1388 nt)	treB ←

References

[1] Hui Wang Ai, Nathan C. Shaner, Zihao Cheng, Roger Y. Tsien, and Robert E. Campbell. Exploration of new chromophore structures leads to the identification of improved blue fluorescent proteins. *Biochemistry*, 46(20):5904–5910, 5 2007.

[2] Rudolf O. Schlechter, Hyunwoo Jun, Michał Bernach, Simisola Oso, Erica Boyd, Dian A. Muñoz-Lintz, Renwick C.J. Dobson, Daniela M. Remus, and Mitja N.P. Remus-Emsermann. Chromatic Bacteria - A Broad Host-Range Plasmid and Chromosomal Insertion Toolbox for Fluorescent Protein Expression in Bacteria. *Frontiers in microbiology*, 9(DEC), 12 2018.

[3] Part:BBa J23119 - parts.igem.org.

[4] Alexander C. Reis and Howard M. Salis. An automated model test system for systematic development and improvement of gene expression models. *ACS Synthetic Biology*, 9(11):3145–3156, 11 2020.

[5] Heng Li. Minimap2: Pairwise alignment for nucleotide sequences. *Bioinformatics*, 34(18):3094–3100, 2018.

[6] Fritz J. Sedlazeck, Philipp Rescheneder, Moritz Smolka, Han Fang, Maria Nattestad, Arndt Von Haeseler, and Michael C. Schatz. Accurate detection of complex structural variations using single-molecule sequencing. *Nature Methods*, 15(6):461–468, 2018.

[7] Daniel E. Deatherage and Jeffrey E. Barrick. Identification of mutations in laboratory-evolved microbes from next-generation sequencing data using breseq. *Methods in molecular biology (Clifton, N.J.)*, 1151:165–188, 2014.

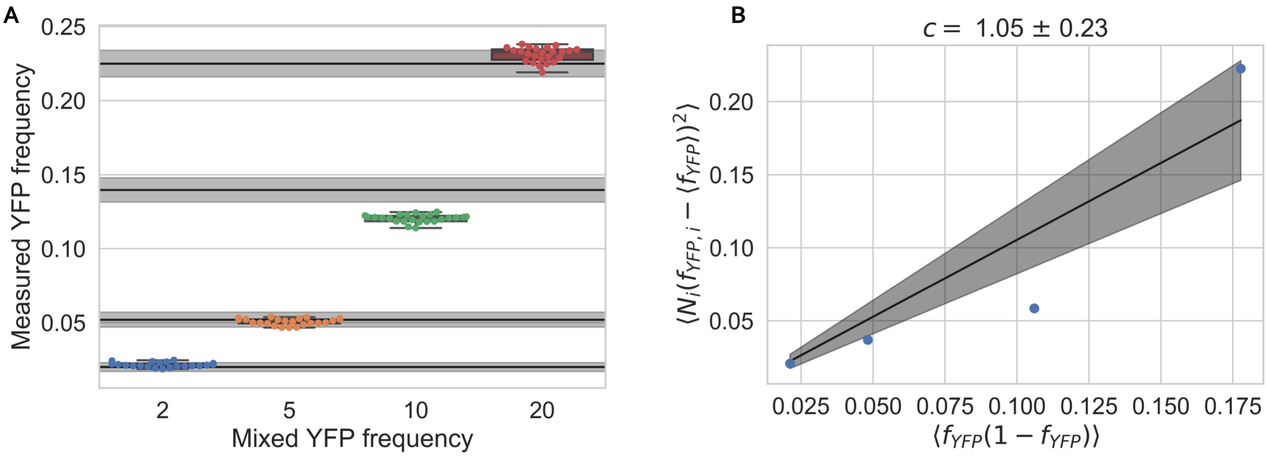

Figure S1: Accuracy of flow cytometer in recovering frequencies of fluorescently-labeled strains in a population. (**A**) We cultured YFP- and BFP-tagged versions of REL606 in DM25, then mixed the cultures at four different frequencies. After culturing for another serial dilution round, we measured each culture 24 times in the flow cytometer (i.e. technical replicates). We also plated the cultures on LB/agar plates and counted the number of blue/yellow colonies for each culture to get CFUs. The colored points represent the flow cytometry measurements. The black lines represent frequencies from CFUs for each of the four cultures, ± standard error. We see that the CFU and flow cytometry measurements mostly match up. (**B**) A plot showing the (scaled) mean-variance relationship, where the variables are transformed such that we would expect a linear relationship with a slope of 1 if the error associated with measuring frequencies followed a binomial, and ≥ 1 if it was overdispersed. $f_{YFP,i}$ represents the relative frequency of YFP cells in the population, for replicate i, and N_i represents the total number of events from the flow cytometer. The blue points are measurements from each experiment, and the line is the fit to the points. Indeed, we see that error looks approximately binomial.

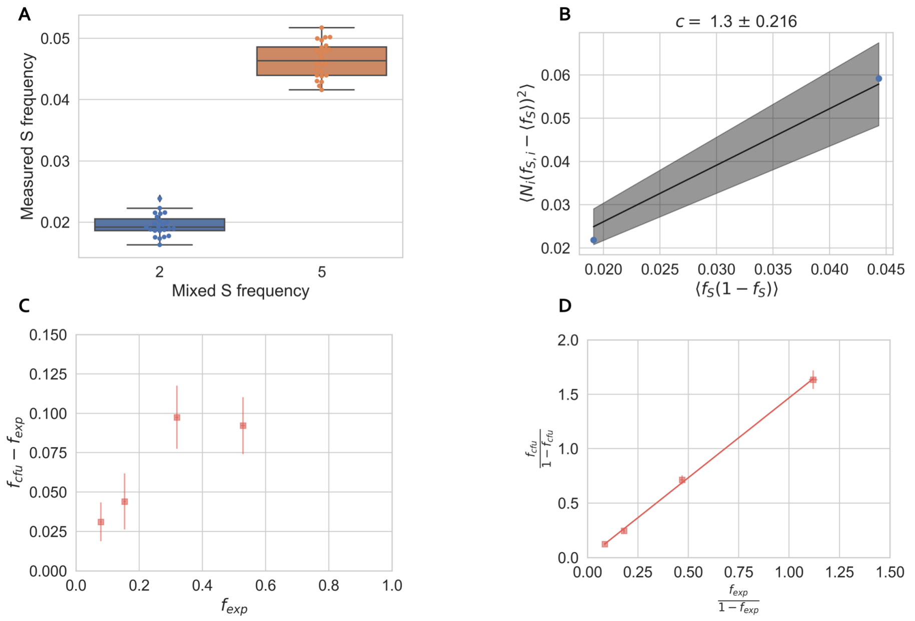

Figure S2: Flow cytometry controls and bias of S/L colony forming units (CFUs). (**A**) We cultured YFP- and BFP-tagged versions of 6.5k L 1 and S 1 respectively in DM25, then mixed the cultures at two different frequencies. After culturing for another serial dilution round, we measured each culture 24 times in the flow cytometer (i.e. technical replicates). The colored points represent the flow cytometry measurements. (**B**) A plot showing the (scaled) mean-variance relationship, similar to Figure S1B. Again, we see that measurement error is consistent with a binomial. (**C**) In a separate experiment, we cultivated four 6.5k L 1 and S 1 cultures at different frequencies, and measured frequencies both by taking a single flow cytometry measurement and by plating the cultures on tetrazolium maltose (TM) plates and counting for CFUs (3 independent plates per culture). We compared the S frequencies from the flow cytometer (f_{exp}) to the average S frequencies from CFUs (f_{cfu}) and see that S is consistently over-represented in the CFU measurements. (**D**) We hypothesized that there might be a multiplicative bias affecting frequencies. If this was true, then the true frequency would be $f_S = n_S/(n_S + n_L)$ where n_i is the count of ecotype i; the biased frequency would be $f_S^{bias} = bn_S/(bn_S + n_L)$ where b is the multiplicative bias. Thus, the odds ratio of the true and biased frequencies should be related linearly, $bf_S/(1 - f_S) = f_S^{bias}/(1 - f_S^{bias})$. We indeed see a linear relationship between the odds ratio of the CFU and flow cytometry frequencies, where $\hat{b} \approx 1.5$. If we trust the flow cytometry measurements more, at least because it more directly measures cell counts and it is unbiased with the LTEE ancestor REL606, then this would imply that measuring S/L frequencies with CFUs incurs a significant multiplicative bias.

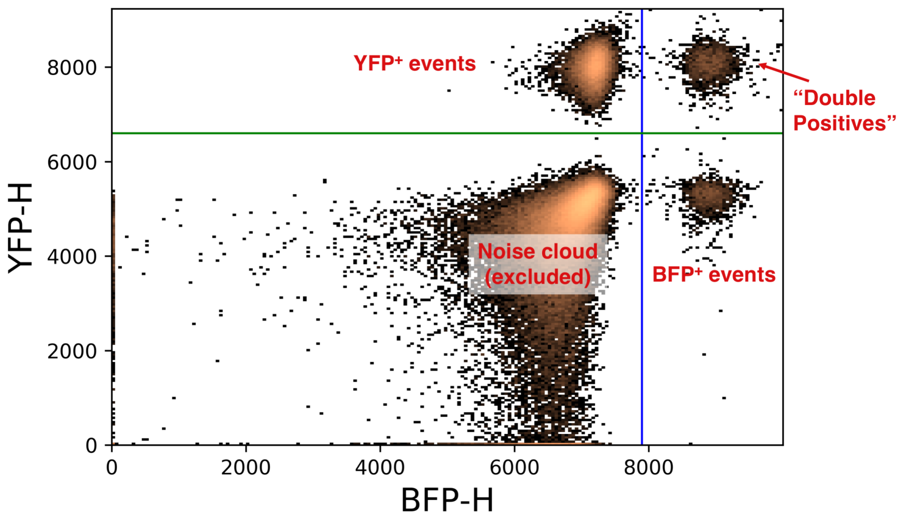

Figure S3: Example of raw flow cytometry data and gates, shown as a 2D histogram of events. The lower left quadrant represents "noise" that is present even when running blank media, and is thus excluded from further analysis. The lower right and upper left quadrants represent events that were called as either BFP or YFP positive, respectively. The upper right quadrant is composed of events that are both BFP and YFP fluorescent; it appears that these events happen when both a BFP and YFP positive cell pass in front of the flow cytometry lasers and are counted as one event. This process appears to be well-described as a poisson process; when we account for this, we appear to recover the correct frequencies (Figure S1). The process by which we account for "double positive" events is detailed in the Methods section.

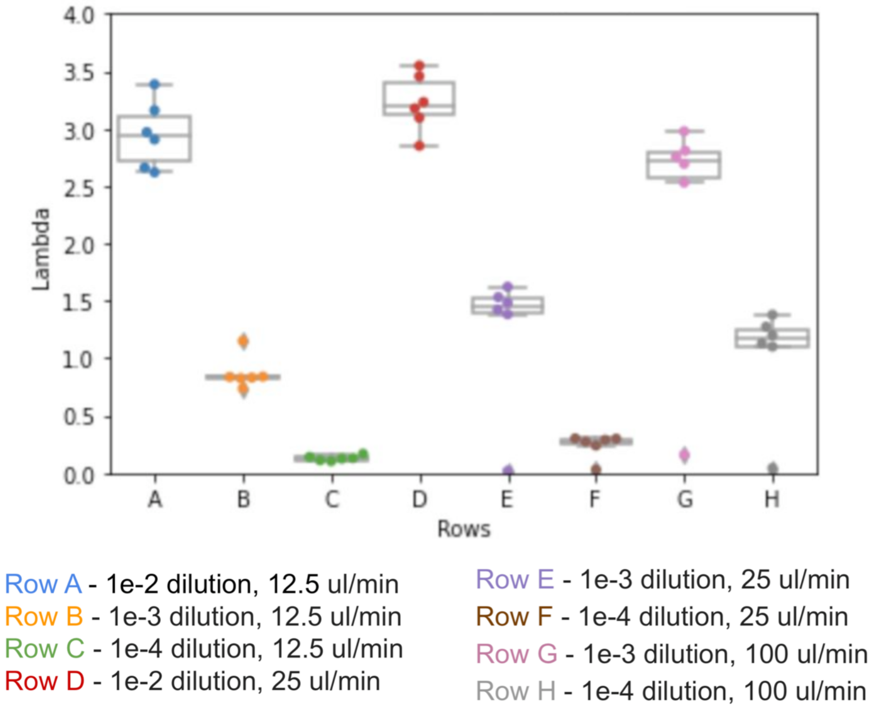

Figure S4: The effect of varying dilution rate and flow rate in the flow cytometer on the average number of cells that end up in front of the laser, i.e. the poisson mean λ.

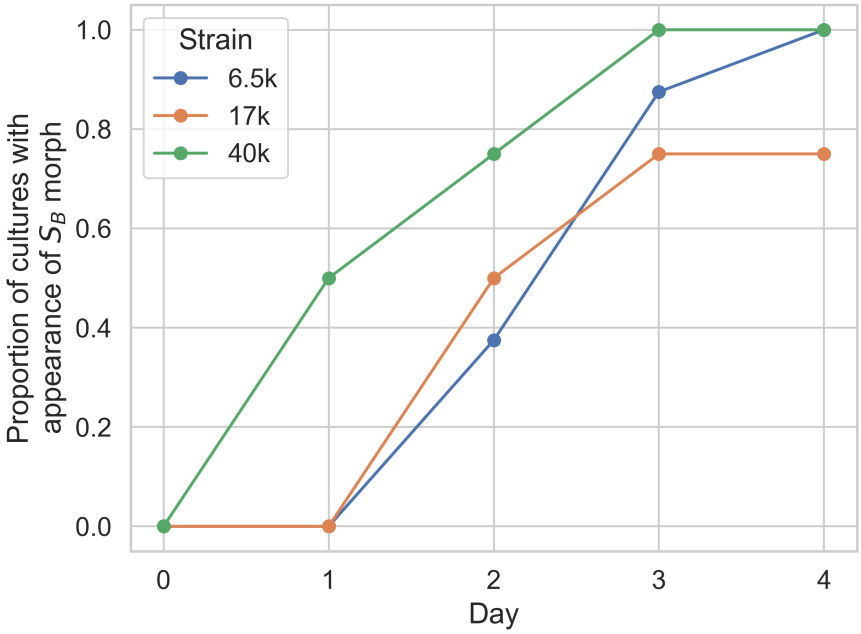

Figure S5: Rediversification of S into S_B in LB. At day 0, we started S clones (picked from DM2000 plates) in 8 replicate 1mL LB cultures, each for S clones from 6.5k, 17k, and 40k LTEE generations. Every day thereafter, we diluted the culture 1:100 into 1mL fresh LB, and we plated the cultures on TA plates (to a final density of around 100 colonies). We then recorded if we saw the appearance of the S_B morph in each of the cultures. We saw the appearance of S_B in most of the cultures across the S clones from the three LTEE timepoints.

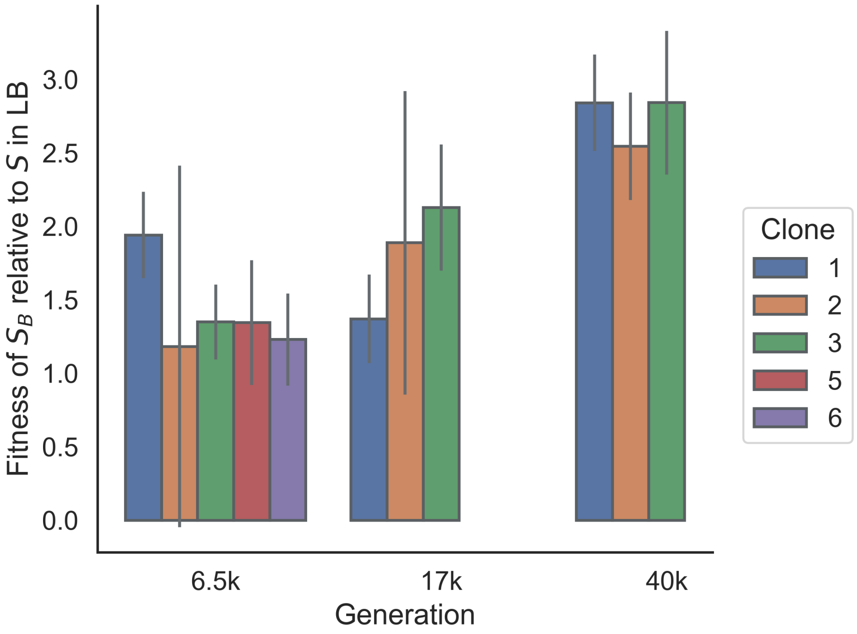

Figure S6: Fitness of S_B clones in LB, relative to S. All S_B and S clones were propagated for one day in DM25, then mixed at approximately a 50-50 ratio, and transferred 1:100 to LB. Frequency of S_B was determined by counting colonies on TA plates. Fitness is computed as the change in logit frequency. Error bars represent standard errors.

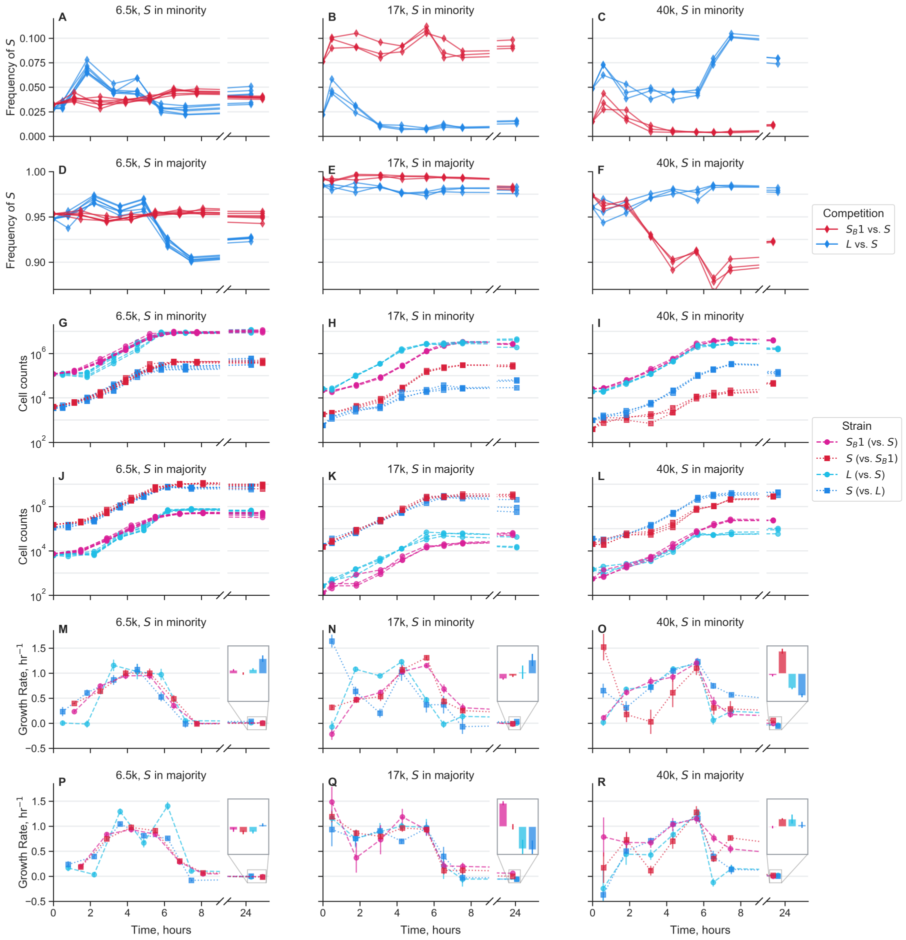

Figure S7: Growth dynamics of cocultures over the course of one twenty-four hour growth cycle. Measurements were taken approximately every hour via flow cytometry for the first eight hours after transfer into new media. An additional measurement was taken approximately 24 hours after the start of the cycle. Mixed S_B with S along with L with S from the same LTEE generation, where ecotypes were mixed both in the majority and minority of the population. All competitions involved S_B used clone 1. Different lines represent biological replicates. Plots in left column represent experiments done with clones from 6.5k generations; 17k clones in the middle column; 40k clones in the right column. (**A-F**) Frequency dynamics of S against S_B and against L. (**G-L**) Total cell count dynamics, separated by each strain in the cocultures. (**M-R**) Growth rates over time for each strain in the cocultures, calculated as the log-slope between adjacent timepoints, using the second timepoint as the x-axis location. Insets represent growth rates in stationary phase, from around 8 to 24 hours. All insets use the same y-axis limits, set from -0.13 to 0.13 hr^{-1}. Error bars represent standard errors.

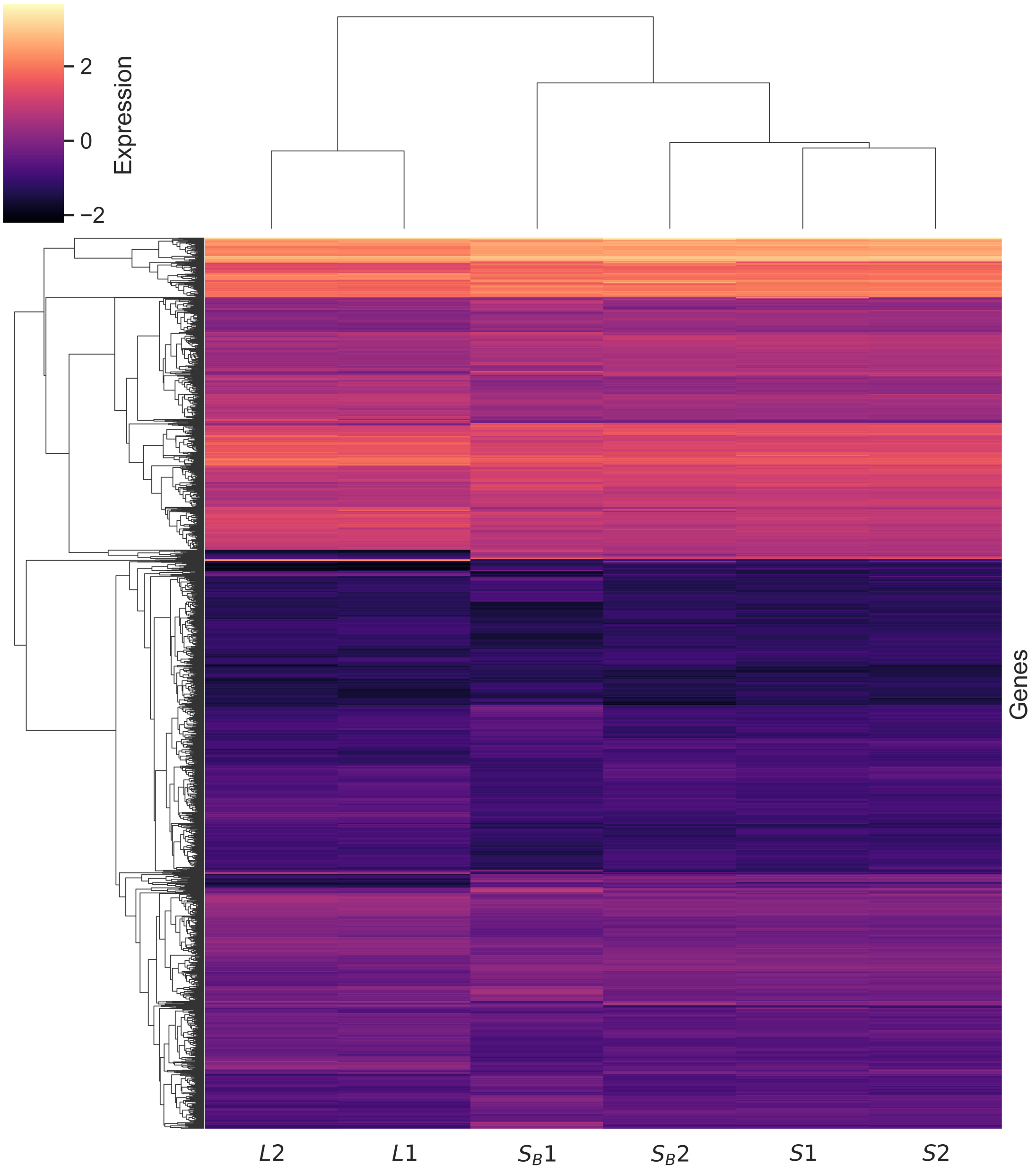

Figure S8: Heatmap and clustering of gene expression patterns. Expression of individual genes is clustered on the y-axis, expression of strains are clustered on the x-axis. We used the variance-stabilized gene expression values from DESeq2, then averaged the two biological replicates for each strain, and centered and scaled the values.

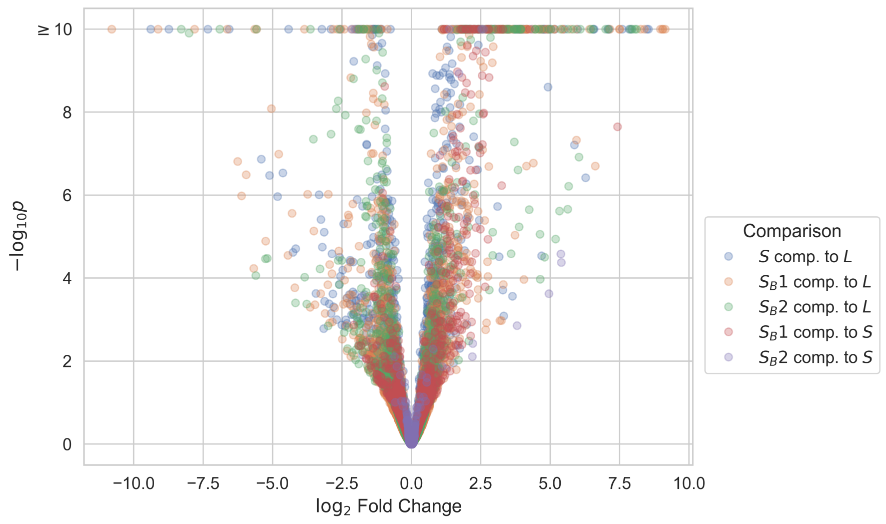

Figure S9: Volcano plot of differential expression, comparing different strains.

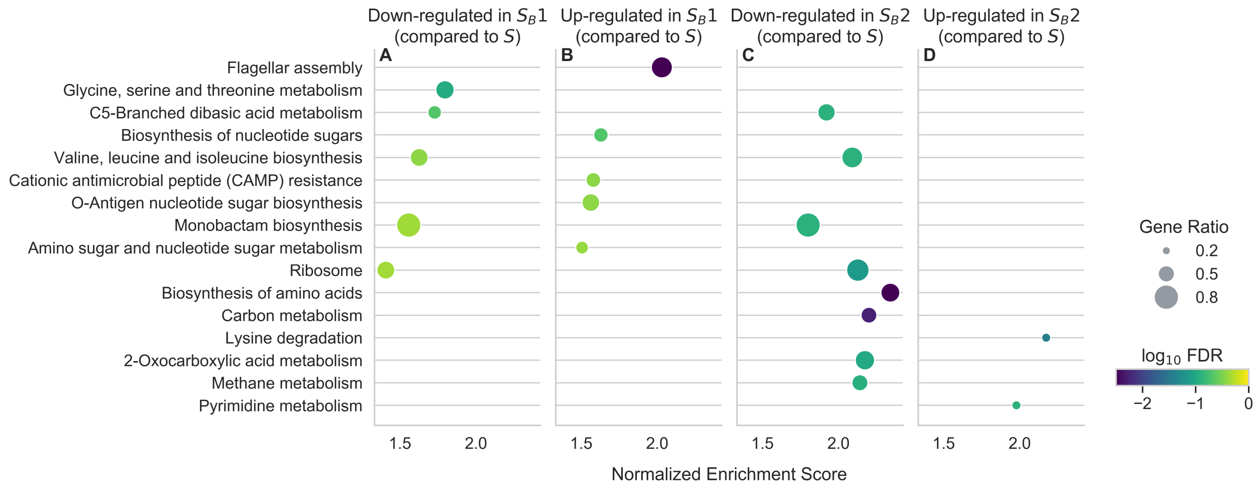

Figure S10: Kegg gene set enrichment analysis for the two S_B clones, showing the top ten genes (sorted by FDR-corrected p-value) for each clone.

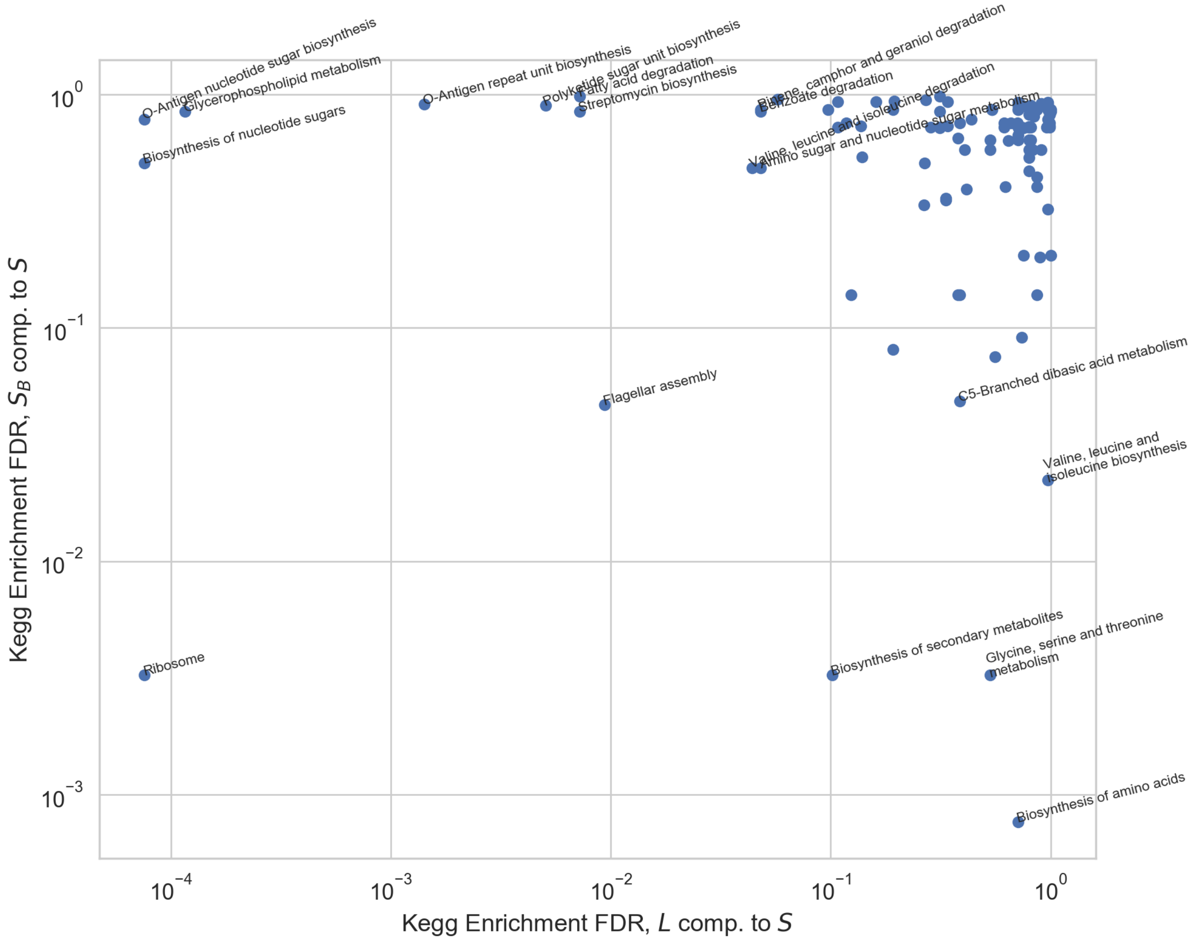

Figure S11: Comparison of differentially expressed KEGG pathways between S_B and L. Shown are the FDR-corrected p-values of each KEGG pathway, with the name shown of significantly enriched pathway in either condition. With the exception of the "Ribosome" and "Flagellar Assembly" pathways, the pathways differentially expressed in S_B and L are mostly orthogonal to each other.

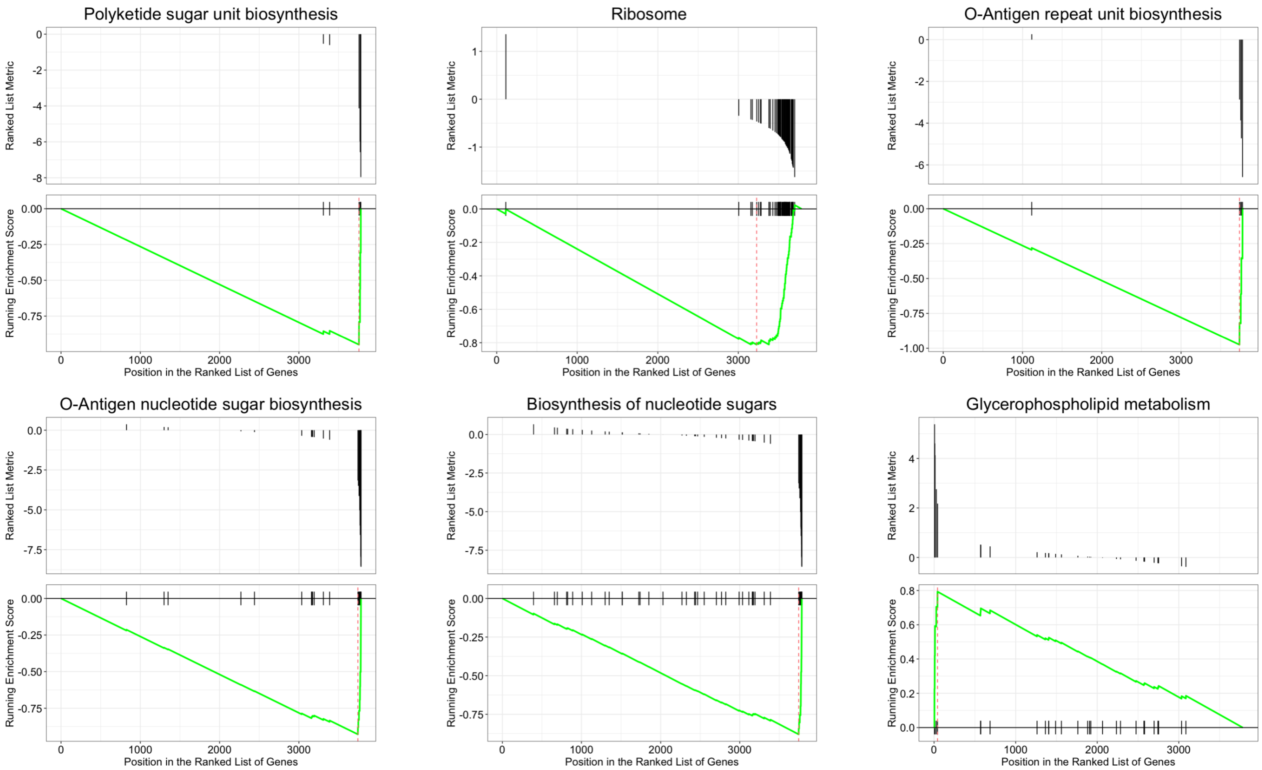

Figure S12: Gene set enrichment analysis plots, comparing L and S. Each subpanel represents a different KEGG pathway that was called as significantly enriched when comparing expression patterns of the two strains. The metric used was $\log_2$ fold change in gene expression (from DESeq2) of L, compared to S.

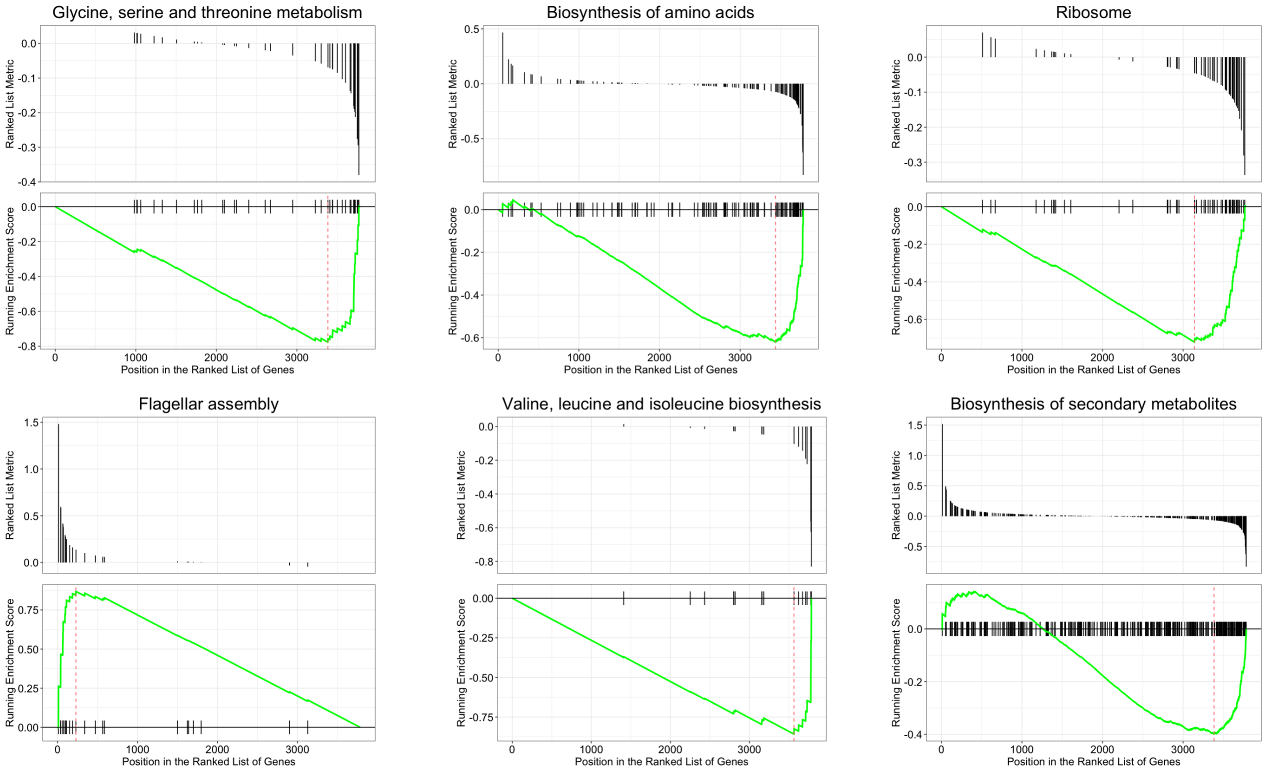

Figure S13: Gene set enrichment analysis plots, comparing S_B and S. Each subpanel represents a different KEGG pathway that was called as significantly enriched when comparing expression patterns of the two strains. The metric used was $\log_2$ fold change in gene expression (from DESeq2) of S_B, compared to S.
